# Simultaneous localization of MLL, AF4 and ENL genes in interphase nuclei by 3D-FISH: MLL translocation revisited

**DOI:** 10.1186/1471-2407-6-20

**Published:** 2006-01-24

**Authors:** Michaël Gué, Jian-Sheng Sun, Thomas Boudier

**Affiliations:** 1 USM 0503, Département "Régulations, Développement et Diversité Moléculaire", Muséum National d'Histoire Naturelle, UMR 5153 CNRS-MNHN, U 565 INSERM, 43 rue Cuvier, CP26, 75231 Paris Cedex 05, France; 2 Institut Curie – Section Recherche, U759 INSERM – Laboratoire d'Imagerie Integrative. Centre Universitaire, Batiment Raymond Latarget, 91405 Orsay CEDEX, France

## Abstract

**Background:**

Haematological cancer is characterised by chromosomal translocation (e.g. MLL translocation in acute leukaemia) and two models have been proposed to explain the origins of recurrent reciprocal translocation. The first, established from pairs of translocated genes (such as *BCR *and *ABL*), considers the spatial proximity of *loci *in interphase nuclei (static "contact first" model). The second model is based on the dynamics of double strand break ends during repair processes (dynamic "breakage first" model). Since the *MLL *gene involved in 11q23 translocation has more than 40 partners, the study of the relative positions of the *MLL *gene with both the most frequent partner gene (*AF4*) and a less frequent partner gene (*ENL*), should elucidate the *MLL *translocation mechanism.

**Methods:**

Using triple labeling 3D FISH experiments, we have determined the relative positions of *MLL*, *AF4 *and *ENL *genes, in two lymphoblastic and two myeloid human cell lines.

**Results:**

In all cell lines, the *ENL *gene is significantly closer to the *MLL *gene than the *AF4 *gene (with P value < 0.0001). According to the static "contact first" model of the translocation mechanism, a minimal distance between *loci *would indicate a greater probability of the occurrence of t(11;19)(q23;p13.3) compared to t(4;11)(q21;q23). However this is in contradiction to the epidemiology of 11q23 translocation.

**Conclusion:**

The simultaneous multi-probe hybridization in 3D-FISH is a new approach in addressing the correlation between spatial proximity and occurrence of translocation. Our observations are not consistent with the static "contact first" model of translocation. The recently proposed dynamic "breakage first" model offers an attractive alternative explanation.

## Background

The Mixed Lineage Leukaemia (*MLL*) gene (also called *HRX*, *HTRX *or *ALL-1*) on chromosome band 11q23 is a recurrent target of reciprocal translocation in human acute leukaemias. The 11q23 translocation occurs in 5–6% of patients with acute myelogenous leukaemia (AML) and 7–10% of patients with acute lymphoblastic leukemia (ALL). More than 40 partners are involved in MLL translocation [2,3]; the most common partner being the *AF4 *gene, on band 4q21. Translocation t(4;11)(q21;q23) is involved in a third of all MLL translocation cases [4] and represents 95% of translocations involved in ALL, compared to only 3.3% in AML [5]. Another frequent partner of translocation is the *ENL *gene located on band 19p13.3, but whilst translocation t(11;19)(q23;p13.3) is found in both ALL and AML, it represents only 6% of all MLL translocations.

It has been demonstrated that pairs of genes (and chromosomal domains) which are frequently involved in reciprocal chromosomal translocation are close to each other in interphase nuclei. These observations have established a direct correlation between the spatial proximity of loci in interphase nuclei and the frequency of their translocation. This implies that the translocation takes place where and when the chromatin fiber containing the partner genes are co-localized (static "contact first" model). However, recent investigations, using alpha-particles for targeting double-strand breaks (DSBs), have demonstrated some displacements of DNA breaks into DNA repair *foci *where chromosomal exchange can occur. In other words, breaks formed at distal locations can subsequently be brought together to induce translocation (dynamic "breakage first" model).

In the present work, we propose a new direct approach to study the correlation between the relative position of genes in interphase nuclei and the corresponding frequencies of translocation. We have applied our approach to genes involved in 11q23 translocations (*MLL*, *AF4 *and *ENL *genes) in differentiated haematological cell lineages by 3D-FISH and in-depth image data analysis.

## Methods

**Figure 1:**
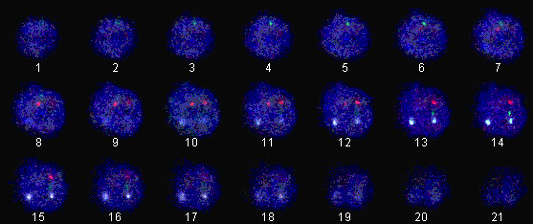
Example of 4-color images from simultaneous 3D-FISH experiments in NALM-6 cell nucleus. Labeled genes were *MLL*, *AF4 *and *ENL *represented by green, white and red spots respectively. Nuclear DNA stained with DAPI is displayed in blue.

**Figure 2:**
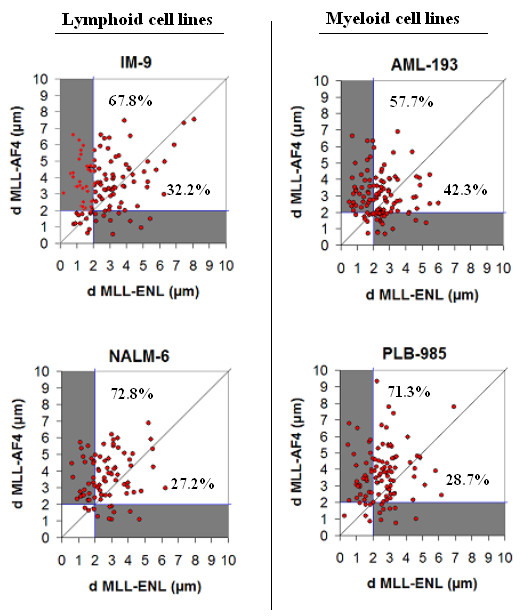
2D – Plot of minimum value of measured distances between *MLL *to *AF4 *and *MLL *to *ENL *genes for all cells. Distances are expressed in μm. Vertical and horizontal blue lines symbolize the gene proximity criterion (2 μm) below which genes could be potentially translocated. Values express the percentage of cells in which the distances reported in one axis is smaller than the distance on the other axis. Grey areas correspond to nuclei where only one of gene could be translocated.

Four hematological cancer cell lines were chosen ; [NALM-6, IM-9, AML-193 and PLB-985] for their human near-diploid karyotype and because the chromosomes of interest (4, 11 and 19) were unmodified. NALM-6 is a bona fide lymphoid leukemia cell line, derived from the peripheral blood of a 19-year-old male with B-precursor acute lymphoblastic leukemia. IM-9 is a non-malignant lymphoid cell line derived by Epstein-Barr virus (EBV) tranformation of residual normal B-cells from a patient with multiple myeloma. AML-193 is a bona fide myeloid leukemia cell line, derived from a 13-year-old female with acute myeloid leukemia, subtype M5. PLB-985 is a false cell line, the result of a cross-contamination with the myeloid leukemia cell line HL-60. Hence cultures labeled as PLB-985 contain in reality HL-60 cells. (All cells were purchased from DSMZ – Braunschweig, Germany – except the NALM-6 cell line which was kindly provided by L. Lagneaux – Belgium). Cells were not synchronized and, in order to preserve their natural nuclear structure, no drugs were added. 3D-FISH was performed according to the standard protocol [16]. Bacterial Artificial Chromosomes (BAC) and Phage Artificial Chromosomes (PAC) were obtained from the Resources for Molecular Cytogenetics database (Italy) [17] : CTD-217A21 for the *MLL *gene and RP11-476C8 for the *AF4 *gene. RP11-2344B19 for the *ENL *gene was purchased from Invitrogen (Carlsbad, California). dUTP-Alexa 488, dUTP-Cy3 and dUTP-Cy5 were used to label BAC/PAC DNA (using random priming protocols) corresponding to *MLL*, *AF4 *and *ENL *genes respectively. Simultaneous hybridization was performed in order to compare the distance between the *MLL *gene and each potential translocation partner inside a single nucleus (figure 1). Confocal microscopy was carried out using the TCS confocal imaging system, equipped with a 63× objective with a numerical aperture set to 1.4 nm. The confocal pinhole was adjusted to allow a minimum field depth. The focus step between sections was 0.35 μm and the XY pixel was set to 100 nm. Gene to gene distances were calculated using Smart 3D-FISH, an ImageJ plugin, recently developed in our laboratory [19]. More than 1000 nuclei were analysed. Only cells (415) displaying two *loci *and adequate morphological preservation of chromatin – as assessed by 4'-,6-diamino-2-phenylindole (DAPI) staining – were retained for analysis. Inter-loci distances were normalized to nuclear diameter: first, the nuclear volume was calculated from the total volume of DAPI stained DNA; a spherical approximation was then used to calculate the diameter of the nucleus from this volume. The minimum value of measured distances was expressed in microns (figure 2) according to gene proximity criterion in which all loci separated by less than 2 μm, could be in contact by Brownian motion prior to the translocation event (according to the "contact first" model). Statistical analysis was performed using Student's *t *test with a level of significance : α = 5%.

## Results and discussion

The mean inter-loci distance measurements between *MLL*, *ENL *and *AF4 *genes in the four cell lines are reported in table 1 (expressed as a percentage of nuclear diameter). No significant overall difference was observed in inter-loci distances between myeloïd and lymphoïd cell lines, despite cell line specific genome organization[21].

In all the studied cell lines, the average separation between MLL and ENL loci was significantly smaller than that observed for MLL and AF4 (P < 0.0001). The mean smallest intergenic distance (corresponding to the minimum value of each of the four inter-loci distances) follows the same tendancy (26% of nuclear diameter for MLL-ENL distance *vs*. 34% for MLL-AF4 distance in NALM-6 and IM-9 cell lines, 26% *vs*. 36% for PLB-985 cell lines and 24% *vs*. 29% for AML-193 cell lines).

For each nucleus, the two smallest intergenic distance – d_MLL-AF4 _and d_MLL-ENL _– are displayed in figure 2. Firstly, our results show that, for the majority of nuclei, the *ENL *gene is closer to the *MLL *gene than to the *AF4 *gene. Secondly, according to the gene proximity criterion, we have calculated the number of nuclei where only the *AF4 *gene should be translocated with *MLL *gene (*i.e. *d_MLL-AF4 _< 2 μm and d_MLL-ENL _> 2 μm, greyed area in figure 2). In the same way, we computed the number of cells in which only the *ENL *gene should be translocated with the *MLL *gene (*i.e. *d_MLL-ENL _< 2 μm and d_MLL-AF4 _> 2 μm, greyed area in figure 2). The ratio of these two values, respectively, indicates a greater probability of a translocation event between *MLL *and *ENL*. (0.33, 0.35, 0.30 and 0.68 for NALM-6, IM-9, PLB-985 and AML-193 cell lines respectively).

Published MLL translocation frequencies report a high level of translocation t(4;11) compared to translocation t(11;19). According to the static "contact first" model, AF4 *loci *should be closer to MLL than ENL loci. Clearly, this is not in accordance with our data. In the case of the 11q23 translocation, the dynamic of chromatin must be taken into account and our results could be the first observation of the dynamic breakage first model proposed by Kanaar and co-workers[12].

It should be noted that published translocation frequencies were established *after *manifestation of cancer. Taking into account that a second genetic event seems to be required to induce cancer [22], we cannot exclude that the chromatin architecture context after MLL-AF4 translocation may be more favourable to the induction of a second event compared to MLL-ENL translocation. This could explain the high level of translocation t(4;11) detected in acute leukaemia. This could be illustrated by the relatively small distances between the ENL gene and the AF4 gene which could be favorable to translocation while the corresponding translocation is never reported in literature[3].

## Conclusion

This work, based on simultaneous multi-probe hybridization in 3D-FISH experiments describes a new approach to study translocation mechanisms in a chromatin context. This method offers the advantage of a direct comparison of several translocation frequencies with localization of corresponding loci in each nucleus. Furthermore, it allows the simultaneous measurement of multiple intergenic distances in a cell by cell manner in a single experiment, without the chromatin distortion common to existing procedures.

Our results are not in accordance with the static model of translocation mechanism and may represent the first observation of the dynamic "breakage first" model. We are currently extending our approach to study MLL and up to six partner genes to confirm these intriguing results.

## Competing interests

The author(s) declare that they have no competing interests.

## Authors' contributions

MG carried out the cytogenetics studies and data analyses

TB participated in the data analyses and coordination of the study

JSS participated in the design of the study

All authors read and approved the final manuscript

## Pre-publication history

The pre-publication history for this paper can be accessed here:



## Figures and Tables

**Table 1:** 

	Cell Lineage [No of loci]			
	
	NALM-6 [n = 324]	IM-9 [n = 448]	PLB-985 [n = 432]	AML-193 [n = 444]
*MLL-ENL*	45.0 ± 1.1%	45.3 ± 1.0%	45.7 ± 0.9%	39.4 ± 0.8%
*MLL-AF4*	54.8 ± 1.1%	55.4 ± 1.0%	57.8 ± 1.0%	46.6 ± 0.9%
*AF4-ENL*	48.5 ± 1.1%	50.6 ± 0.9%	43.7 ± 0.8%	49.5 ± 1.0%

Mean inter-loci distances between the *MLL*, *AF4 *and *ENL *genes in NALM-6, IM-9, PLB-985, AML-193 cell lines (expressed as a percentage of nuclear diameter).
